# Vertical transmission of HIV among pregnant women who initially had false–negative rapid HIV tests in four South African antenatal clinics

**DOI:** 10.1371/journal.pone.0226391

**Published:** 2019-12-20

**Authors:** Simnikiwe H. Mayaphi, Desmond J. Martin, Thomas C. Quinn, Anton C. Stoltz

**Affiliations:** 1 Department of Medical Virology, University of Pretoria, City of Tshwane, South Africa; 2 National Health Laboratory Service–Tshwane Academic Division (NHLS–TAD), City of Tshwane, South Africa; 3 Toga Laboratories, Johannesburg, South Africa; 4 Division of Intramural Research, National Institute of Allergy and Infectious Diseases, National Institutes of Health, Bethesda, Maryland, United States of America; 5 Johns Hopkins University School of Medicine, Baltimore, Maryland, United States of America; 6 Division of Infectious Diseases, Department of Internal Medicine, University of Pretoria, City of Tshwane, South Africa; Yeshiva University Albert Einstein College of Medicine, UNITED STATES

## Abstract

**Introduction:**

There is a risk of mother-to-child transmission of HIV (MTCT) during pregnancy and breastfeeding. The aim of this study was to assess vertical transmission of HIV among pregnant women who initially had false–negative rapid HIV tests in South African antenatal care (ANC) clinics.

**Methods:**

Pregnant participants were enrolled in a diagnostic study that used nucleic acid amplification testing (NAAT) to screen for early HIV infection among individuals who tested negative on rapid HIV tests used at the point-of-care (POC) facilities. Participants were enrolled from four ANC clinics in the Tshwane district of South Africa. All NAAT-positive participants were recalled to the clinics for further management. Vertical transmission was assessed among exposed infants whose HIV polymerase chain reaction (PCR) results were available.

**Results:**

This study enrolled 8208 pregnant participants who tested negative on rapid HIV tests between 2013 and 2016. Their median age was 26 years (interquartile range [IQR]: 23–30). NAAT detected HIV infections in 0.6% (n = 49; 95% confidence interval {CI}: 0.5–0.8) of all study participants. The distribution of these infections among the four clinics ranged from 0.3%– 1.1%, but this was not statistically significant (p = 0.07). Forty-seven participants (95.9%) were successfully recalled and referred for antiretroviral treatment initiation as part of prevention of MTCT (PMTCT). Most women with newly diagnosed HIV infection presented for the first ANC visit in the second (61.9%, n = 26) and third (31.0%, n = 13) trimesters. HIV PCR results were available for thirty-two infants, three of whom tested positive (9.4%; 95% CI: 1.98–25.02).

**Conclusions:**

This study showed that supplemental HIV testing for pregnant women led to earlier linkage to the PMTCT programme. Inaccurate diagnosis of HIV infection at ANC clinics is likely to undermine the efforts of eliminating MTCT particularly in HIV-endemic settings.

## Introduction

HIV infection in women of childbearing age is associated with a risk of transmission to infants during pregnancy and breastfeeding. Without intervention, the risk of vertical transmission is highest during the intrapartum period and breastfeeding, but lower in the antepartum period [1]. Prevention of mother-to-child transmission of HIV (PMTCT) with antiretroviral (ARV) drugs has remarkably reduced the rate of vertical transmission [2], and this has led to elimination of MTCT (eMTCT) in some countries [3].

PMTCT guidelines have evolved over time from use of a single drug during pregnancy [4, 5] to recommending highly active antiretroviral therapy (HAART). Option B+ strategy refers to immediate initiation of life-long HAART among HIV-infected pregnant or breastfeeding women regardless of their CD4 count level, and is associated with higher efficacy for reduction of MTCT [1, 6]. South Africa (SA) implemented the option B+ strategy in March 2013 [7], and has been making a good progress in reducing MTCT even before the introduction of this strategy [8–10]. However, more efforts are needed for achieving eMTCT in SA as a recent study reported a rate of 245 in-utero paediatric HIV infections per 100 000 live births nationally [11].

HIV screening during pregnancy plays an important role in identifying HIV-infected individuals who would benefit from the PMTCT programme. Unfortunately, some pregnant women with HIV infection receive false-negative rapid tests at SA point-of-care (POC) facilities [12–14]. SA studies have reported false-negative rapid tests due to HIV sero-conversion during pregnancy, with rates ranging between 0.9–3.4% [13, 15, 16]. Recent HIV infection during pregnancy is associated with a higher risk of MTCT [16, 17]. Black et al, demonstrated lower field sensitivity of rapid tests in an antenatal clinic (ANC) based at a tertiary hospital in Johannesburg, highlighting that some pregnant women with chronic HIV infection are misdiagnosed [12]. The aim of this study was to assess vertical transmission of HIV among pregnant women who initially had false-negative rapid HIV tests in SA ANC clinics.

## Materials and methods

### Participants’ enrolment

This study included pregnant women who participated in a previously published diagnostic study that screened for early HIV infection among individuals who tested negative on rapid HIV tests used at the POC facilities [14], and those who were enrolled after the publication time. Those who were diagnosed with HIV infection were prospectively followed up for further testing and linkage to care. Clinic records of study participants were later reviewed during or after completion of enrolment. Enrolment period was from March 2013 –December 2016, which is the time SA had implemented the option B+ strategy [7]. Participants were enrolled after HIV testing during the first ANC visit from four clinics (FF Ribeiro, Skinner, Stanza Bopape and Phomolong) based in different areas of the Tshwane district of SA. Recruitment and enrolment of study participants was done at the clinics by research assistants who had received training in HIV counseling and testing (HCT) and had been providing HCT at the clinics prior to their involvement in this study. SA adopted a serial testing strategy for HIV testing at POC facilities, where a screening rapid test is performed first, and confirmatory testing is only performed if the screening test is positive [18]. The rapid HIV test that was commonly used for screening during the course of this study was Advanced Quality (Intec Products Inc). Abon (Abon Biopharm) rapid test was used for screening in 2014; however, this was replaced with Advanced Quality at the end of 2014.

### Study samples and testing procedures

At enrolment, study samples and participants’ cell phone numbers were obtained. Plasma was separated from whole blood through centrifugation and stored at -70ºC within 24 hours after collection. Pooled nucleic acid amplification testing (NAAT) was performed using Roche COBAS AmpliPrep/COBAS TaqMan HIV-1 VL version 2 assay (Roche Diagnostics, Mannheim, Germany) in mini-pools of 5 samples, followed by individual sample NAAT in positive pools. All NAAT-positive women were recalled to the clinics for appropriate management, which included confirmatory HIV testing and referral for immediate initiation of HAART according to SA PMTCT guidelines [7].

Further serology tests were performed in NAAT-positive samples. These included 3^rd^ generation Genscreen HIV-1/2 version 2 ELISA (BioRad, Marnes-la-Coquette, France) and HIV Western Blot (Bio-Rad Laboratories, Redmond WA, USA) for antibody detection; p24 antigen (Roche Diagnostics, Mannheim, Germany); and limiting antigen (LAg) HIV avidity assay {Maxim Biomedical Inc., Rockville, USA} for confirmation of early HIV infection in samples with detectable antibodies. Acute HIV infection was diagnosed in participants who had detectable HIV RNA with or without p24 antigen in the absence of HIV antibodies. Early HIV infection was diagnosed in participants who had detectable HIV RNA with or without p24 antigen, and presence of HIV antibodies with low avidity as reflected by values <1.5 normalised optical density (OD-n) on LAg avidity assay. Participants who had detectable HIV RNA with or without p24 antigen, and presence of HIV antibodies with high avidity (>1.5 OD-n) were diagnosed with chronic HIV infection [14]. False-negative rapid tests during acute or early HIV infection (sero-conversion) are associated with absence or lower levels of antibodies. However, false-negative rapid tests during chronic HIV infection likely represent misdiagnosis due to errors during testing, as free antibodies are expected to be detectable in this phase [19, 20].

### Pregnancy and PMTCT data collection

Data on gestation at first ANC visit, initiation of PMTCT and mode of delivery were later collected from the mothers’ clinic records. Between 2013 and 2015, SA HIV testing strategy for pregnant women who tested negative at the first ANC visit recommended a repeat test every 3 months throughout pregnancy, at delivery, at 6 weeks post-partum, and 3 monthly throughout breastfeeding [7, 21]. These guidelines, however, changed in 2016 to recommend HIV retesting at every ANC visit, at delivery and every 3 months during breastfeeding [18].

Data on infant’s names, date of birth, receipt of HIV prophylaxis, feeding method and HIV polymerase chain reaction (PCR) results were collected from the infants’ clinic records. Where it was difficult to locate clinic records, mothers were phoned for some information such as names and date of birth, and this was used to search for infant’s HIV PCR results from the laboratory information system. Infants were follow-up to 18 months of age. During the study period, SA HIV management guidelines for infants born to HIV-infected mothers recommended exclusive breastfeeding [7, 21]. These guidelines recommended the first HIV PCR test at birth (implemented from June 2015), with follow-up PCR tests done at 4–6 weeks after completing HIV prophylaxis, 6 weeks after cessation of breastfeeding, or whenever there is an indication before 18 months of age. Rapid HIV test was recommended from 18 months onwards [21]. There was no birth HIV PCR results for infants born before June 2015 as previous guidelines recommended the first HIV PCR test at 6 weeks of age, follow-up PCR test at 6 weeks after cessation of breastfeeding, or HIV PCR test whenever there was an indication before 18 months of age [7].

### Ethics

The study was approved by the University of Pretoria Faculty of Health Sciences Ethics Committee (Protocol number–295/2015) and by Tshwane–Metsweding Region Research Ethics Committee (TMREC 2010/26). The legal ages for consenting to HIV testing and medical treatment in SA are 12 and 14 years, respectively [22]. This study enrolled participants aged 14 years or older. The initial study protocol was approved for participants older than 18 years but it was later amended and approved to include those from 14 years of age to extend the benefits of screening for early HIV infection to younger women, most of whom came to the clinics alone. All the study participants agreed to participate and signed written consent forms before enrolment.

### Data analysis

Descriptive statistics was used to present median and interquartile range (IQR), and 95% confidence interval [CI] was computed for the point prevalence estimates. Fisher’s exact test was used to assess association between HIV infections and study clinics. A p-value of ≤0.05 was considered for statistical significance. All the statistics were performed on the STATA version 15.1 software (StataCorp LP, College Station, TX, USA).

## Results

### Demographics and newly diagnosed HIV infections

There were 8208 pregnant participants enrolled in this study, from March 2013 –November 2016, with a median age of 26 years (IQR: 23–30). All participants had tested negative on rapid HIV tests used at the POC facilities. Enrollment varied among the four clinics, with majority of participants (51.8%) enrolled from FF Ribeiro clinic. NAAT detected HIV infections in 0.6% (n = 49, 95% CI: 0.5–0.8) of all study participants. The distribution of these infections among the four clinics ranged from 0.3%– 1.1%, but this was not statistically significant (p = 0.07) (Table 1). Thirteen participants (26.5%) had early HIV infection and all others had chronic infection [14] (S1 Table).

**Table 1:** Distribution of participants and HIV infections by clinics.

Clinic name	Participants	HIV infections	95% CI	P-value
	n (%)	n (%)		
FF Ribeiro	4255 (51.8)	25 (0.6)	0.4–0.9	0.07
Skinner	1439 (17.5)	7 (0.5)	0.4–1.9	
Stanza Bopape	1324 (16.1)	14 (1.1)	0.9–3.5	
Phomolong	1190 (14.5)	3 (0.3)	0.1–1.4	

n–sample size, CI–confidence interval

**Fig 1:**
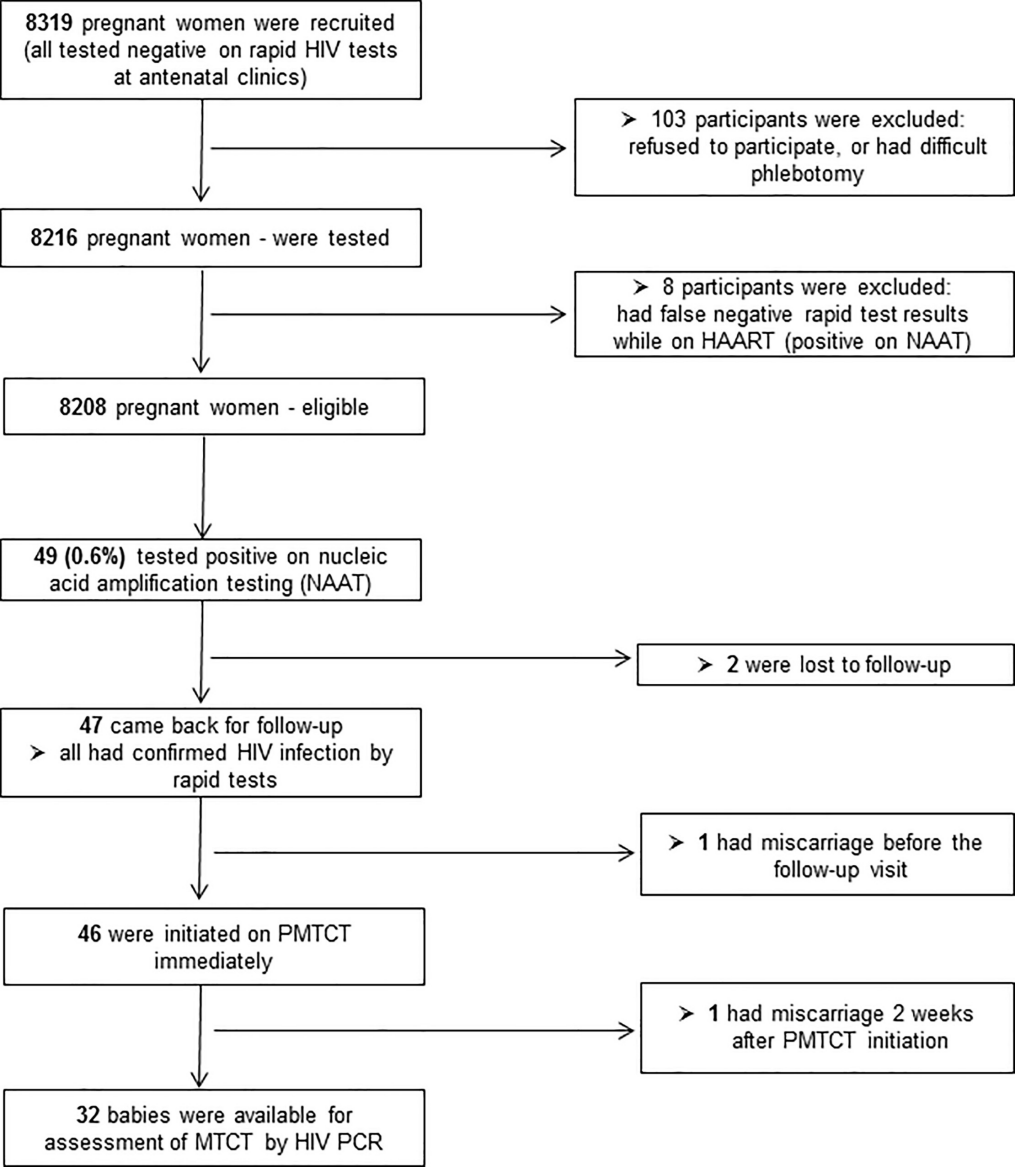
Algorithm showing enrollment of study participants. Those who tested positive on nucleic acid amplification testing (NAAT) were recalled for HIV confirmatory test and immediate initiation of highly active antiretroviral therapy (HAART) as part of prevention of mother-to-child transmission of HIV (PMTCT). Lost to follow-up was mainly due to relocation to other clinics within South Africa and neighbouring countries. PCR = polymerase chain reaction.

All NAAT-positive participants were recalled to their respective clinics for further management, and came at different intervals owing to their availability. Forty-seven participants (95.9%) were successfully recalled to the clinics and referred for PMTCT, except for one participant who had miscarriage (Fig 1). The latter was referred for appropriate management, which at the time (in 2014) recommended initiation of HAART at CD4 count <350 cells/μl [23].

### Pregnancy and PMTCT data

Data on pregnancy was available for 42 women who were initiated on PMTCT programme. Clinic records for four other women could not be accessed, and these participants could also not be contacted by telephone. Most women with newly diagnosed HIV infection presented for the first ANC visit in the second (61.9%, n = 26) and third (31.0%, n = 13) trimesters. Only two participants were still in the first trimester at the time of PMTCT initiation. The median delay between initial negative rapid HIV test and PMTCT initiation was 3 weeks (IQR: 2–4) (Table 2). Even though some women presented earlier for ANC and were contacted after positive NAAT results, they only came back very late for a follow-up visit (Table 2 –#5, 6 and 25). Available data on the mode of delivery and infant feeding showed that most women had vaginal delivery and exclusively breastfed their infants (Table 2). Unfortunately, some women were lost to follow-up after PMTCT initiation owing to various reasons such as relocating to other clinics or neighbouring countries.

**Table 2:** Data on antenatal care and assessment of mother-to-child transmission of HIV.

	MOTHERS' DATA					INFANTS' DATA								
#	Pt	Gestation	Rapid	HIV VL	Gestation	Birth time	Delivery	Nevirapine	Feeding	HIV PCR results				Rapid
	ID	at 1^st^ ANC	HIV test	(copies/ml)	at PMTCT initiation	mmm-yy	mode	prophylaxis	method	0–5 days	6–8 weeks	3–6 months	7–18 months	HIV test
1	5067	19	NEG	1.2 x 10^4^	22	Sep-13	NVD	Yes	BF	--	--	--	--	--
2	9915	30	NEG	1.4 x 10^4^	32	Sep-13	NVD	Yes	BF	--	NEG	NEG	--	--
3	639	16	NEG	6.5 x 10^3^	19	Jun-14	NVD	Yes	BF	--	NEG	--	NEG	NEG
4	641	13	NEG	7.0 x 10^4^	15	Jul-14	--	Yes	BF	--	--	--	--	--
5	6638	24	NEG	1.9 x 10^5^	33	Apr-14	NVD	Yes	BF	--	NEG	--	NEG	NEG
6	8828	25	NEG	4.1 x 10^4^	37	Jul-14	NVD	Yes	BF	--	NEG	NEG	NEG	NEG
7	2678	16	NEG	2.2 x 10^5^	20	Sep-14	C/S	Yes	BF	--	NEG	--	NEG	NEG
8	9895	9	NEG	4.8 x 10^3^	18	Sep-14	--	--	BF	--	--	NEG	NEG	NEG
9	9986	9	NEG	9.7 x 10^4^	11	Dec-14	C/S	--	--	--	NEG	NEG	--	--
10	843	21	NEG	2.9 x 10^4^	24	Oct-14	NVD	Yes	BF	--	NEG	NEG	--	--
11	6512	15	NEG	1.7 x 10^3^	19	Dec-14	--	--	--	--	NEG	--	--	--
12	6990	6	NEG	1.7 x 10^4^	11	--	--	--	--	--	--	--	--	--
13	6671^¥^	17	NEG	1.4 x 10^4^	20	Jan-15	NVD	--	BF	--	--	POS x2	--	n/a
14	6380	21	NEG	1.1 x 10^4^	24	Feb-15	NVD	Yes	BF	--	NEG	--	NEG	NEG
15	6557	22	NEG	6.1 x 10^2^	26	Feb-15	NVD	Yes	BF	--	NEG	--	--	--
16	6565	25	NEG	5.6 x 10^3^	27	Jan-15	NVD	Yes	BF	--	NEG	--	NEG	--
17	6582	31	NEG	6.2 x 10^3^	36	Dec-14	NVD	Yes	BF	--	NEG	--	--	--
18	6596	32	NEG	3.8 x 10^3^	35	Dec-15	NVD	Yes	BF	--	NEG	--	--	--
19	6640	22	NEG	3.0 x 10^3^	26	--	--	--	--	--	--	--	--	--
20	6727	20	NEG	4.8 x 10^3^	23	Feb-15	--	Yes	--	--	NEG	--	--	--
21	6649	15	NEG	2.1 x 10^4^	17	--	--	--	--	--	NEG	--	--	--
22	6738	24	NEG	1.5 x 10^5^	26	--	--	--	--	--	--	--	--	--
23	1067*	24	NEG	1.7 x 10^3^	26	--	--	--	--	--	--	--	--	--
24	2504	19	NEG	3.7 x 10^4^	21	--	--	--	--	--	--	--	--	--
25	921	36	NEG	9.7 x 10^3^	post delivery	Apr-15	C/S	--	--	--	NEG	--	NEG	--
26	3869	32	NEG	2.1 x 10^5^	36	May-15	NVD	--	BF	--	--	--	NEG x2	--
27	3912	21	NEG	3.2 x 10^4^	23	Sep-15	NVD	Yes	FF	--	NEG	--	--	--
28	3920^¥^	23	NEG	6.6 x 10^4^	25	Aug-15	--	--	--	--	--	--	POS x2	n/a
29	3880	22	NEG	7.5 x 10^3^	26	Aug-15	NVD	Yes	BF		NEG	--	--	--
30	3935^¥^	32	NEG	2.4 x 10^5^	34	Jun-15	NVD	Yes	BF	POS	--	POS	--	--
31	1117	24	NEG	1.5 x 10^2^	26	Sep-15	NVD	Yes	BF	NEG	NEG	--	--	--
32	1121	30	NEG	8.0 x 10^4^	32	Jun-15	NVD	Yes	BF	NEG	--	--	--	--
33	3469	23	NEG	3.3 x 10^4^	25	--	--	--	--	--	--	--	--	--
34	3474	28	NEG	1.6 x 10^4^	32	--	--	--	--	--	--	--	--	--
35	1475	27	NEG	4.4 x 10^4^	29	Aug-15	--	--	--	NEG	NEG	--	--	--
36	3387	27	NEG	7.9 x 10^4^	37	Oct-15	NVD	Yes	BF	NEG	--	NEG	--	--
37	3253	13	NEG	8.9 x 10^4^	15	Feb-15	--	--	--	NEG	--	--	--	--
38	1692	16	NEG	3.9 x 10^2^	18	Jan-16	C/S	Yes	FF	--	--	--	NEG	NEG
39	3606	28	NEG	1.5 x 10^4^	32	--	--	--	--	--	--	--	--	--
40	2866	21	NEG	3.3 x 10^3^	24	Jan-16	--	--	--	--	--	NEG	--	--
41	1213	24	NEG	3.2 x 10^4^	26	Aug-16	NVD	Yes	BF	NEG	NEG	NEG	--	--
42	3910	13	NEG	2.9 x 10^4^	16	Jan-17	C/S	Yes	FF	NEG	NEG	--	NEG	--
43	6748†	--	NEG	9.3 x 10^2^	--	--	--	--	--	--	--	--	--	--
44	5054†	--	NEG	2.7 x 10^4^	--	--	--	--	--	--	--	--	--	--
45	9049†	--	NEG	1.6 x 10^4^	--	--	--	--	--	--	--	--	--	--
46	4351†	--	NEG	2.6 x 10^3^	--	--	--	--	--	--	--	--	--	--

Some participants were lost to follow-up at different stages of ANC or post-natal care owing to relocation to other clinics within or outside South Africa. HIV viral load (VL) testing was performed from samples obtained at enrolment (i.e. after a negative rapid HIV test). #—participant (pt) numbering, ID–study identity, 1^st^–first, ANC–antenatal care, ml–millilitre, mmm-yy (month and year), PMTCT–prevention of mother-to-child transmission of HIV, PCR–polymerase chain reaction, NVD–normal vaginal delivery, C/S–Caesarian section, BF–breastfeeding, FF–formula feeding, NEG–negative, POS–positive, --–no data, x2 –two results available in a testing interval. Participants who transmitted HIV to their infants are highlighted in light gray rows.

†–participants were referred for PMTCT initiation during the follow-up visit but their clinic records could not be accessed later, and they could not be contacted by telephone.

*–participant had miscarriage two weeks after HAART initiation.

^¥^The three women who transmitted HIV infection to their infants gave birth between 37–45 weeks gestation; this was estimated from the dates of the first ANC and dates of infants’ birth.

### Assessment of vertical transmission of HIV

Thirty-two infants were successfully followed up to assess vertical transmission (Fig 1). Nevirapine prophylaxis was documented for those whose files were accessible. Most infants (60%, n = 18) had more than one HIV PCR results. HIV infection was diagnosed in three infants (9.4%; 95% CI: 1.98–25.02), all of whom were delivered at term. One infant had a positive HIV PCR three days after birth. It was difficult to estimate the time of HIV transmission in the other two infants as their mothers had relocated to other clinics after PMTCT initiation, and they had no birth HIV PCR results. It is likely that these two infants were symptomatic at the time of HIV testing as their first HIV PCR results fall outside the recommended testing intervals (Table 2). Interestingly, all the mothers who transmitted HIV to their infants had chronic HIV infection, with viral loads ranging from 1.4 x 10^4^–2.4 x 10^5^ copies / milliliter before PMTCT initiation (Table 2) [14]. Rapid HIV tests were not available for most infants as these results are often documented on the clinic cards that are kept by their mothers. Missing information on infants’ HIV PCR tests was mainly caused by loss to follow-up of their mothers, who had relocated to other clinics within SA and neighbouring countries. For instance, some participants relocated to neighbouring countries immediately after giving birth (Table 2 –#1 and #4) and could not be contacted by telephone.

## Discussion

### Missed HIV infections

This study identified HIV infections among pregnant women who initially had false-negative rapid HIV tests at four ANC clinics in the Tshwane district of SA. The finding of 0.6% newly diagnosed HIV-infected individuals at ANC clinics highlights gaps in the quality of HIV testing at the POC facilities. The extent of this problem is reflected by detection of missed HIV infections in all the study clinics. Even though one clinic had a higher rate of HIV infections, this was not statistically significant. Uneven distribution of HIV infections within a province or district has been previously reported in SA [24]. Other studies, including SA studies, have also reported false-negative rapid HIV tests during pregnancy [12, 13, 15, 16, 25–27]. One SA study reported as high as 8.6% of women who had negative rapid test during ANC but were positive during the post-natal period based on infant HIV ELISA [28]. This calls for adequate implementation of the recent South African guidelines on repeat HIV testing during pregnancy to detect both misdiagnosis and sero-conversion. These guidelines recommend retesting at every ANC visit for women who tested negative for HIV at the first ANC visit [18]. PMTCT initiation was delayed in the study participants as a result of inaccurate HIV diagnosis (Table 2).

### False-negative rapid tests during acute or early HIV infection

Most rapid HIV tests only detect antibodies; hence it is not surprising to have negative rapid tests during acute HIV phase, as the antibodies have not developed yet in this stage. The appearance of antibodies (sero-conversion) occurs after about 3 weeks following HIV infection or later. Following sero-conversion during early HIV infection, antibodies often exist in lower levels in the blood and predominantly circulate as antigen-antibody complexes [19, 20]. This is a likely explanation for false-negative rapid tests in participants with early HIV infection whose antibodies were detected by HIV ELISA in plasma samples (S1 Table). Other SA studies have reported on recent HIV infections during pregnancy [13, 15, 16, 25].

### False-negative rapid tests during chronic HIV infection

The two rapid HIV tests used in this study had satisfactory performance during WHO evaluation on a similar panel of serum / plasma samples, with Advanced Quality showing 100% sensitivity and specificity, while the Abon rapid test had 100% sensitivity and 99.7% specificity [29]. A study that screened for acute HIV infections in non-pregnant population in the KwaZulu-Natal province of SA also found a high rate of chronic HIV infections that were missed by rapid tests at the POC facilities [30]. False-negative rapid HIV tests at the POC facilities could be attributed to errors that occur during testing, which might be due to the use of incorrect volume of blood or test diluent [31]. Some studies have demonstrated lower performance of rapid HIV tests on finger-stick whole blood compared to testing on serum samples [12, 32]. In a field evaluation of three rapid HIV tests conducted among SA pregnant women, Black et al. reported a lower sensitivity for all these tests, ranging from 87.5–94.5% [12]. Similar findings were observed in a laboratory-based study that compared rapid HIV tests performance between whole blood and serum sample pairs among non-pregnant individuals [32]. The lower performance of some rapid HIV tests in finger-stick whole blood could be attributed to the following factors; dilution effect from the red blood cells, and weak antibody binding due to haemolysis [32]. Antigen-antibody complexes are predominantly found in early HIV phase owing to high viraemia; this may cause false-negative rapid tests as free antibodies appear later [19]. The antigen-antibody complexes are also found in later stages of HIV infection [33, 34]. Currently, there are no data that suggest that pregnancy is a risk factor for HIV misdiagnosis as other studies have found misdiagnosed infections in non-pregnant populations [14, 30, 35]. Our previous study that assessed HIV risk factors in this cohort did not identify any factors that were significantly associated with HIV misdiagnosis during pregnancy [36].

### Gestation at first ANC visit and risk of MTCT

The finding that most women presented for the first ANC visit in the second and third trimesters is a trend that has been observed by a previous SA study [28]. This shows that most infants were exposed to HIV in-utero for a considerable amount of time before PMTCT was initiated. Of the three women who transmitted HIV to their infants, two presented for the first ANC in the second trimester and one in the third trimester (Table 2). Late booking for ANC is associated with a higher risk of MTCT [37], and has been reported by previous SA studies [38–40]. In the absence of intervention, the risk of vertical transmission of HIV during ANC period is between 20–25% in the first 28 weeks of pregnancy, but increases to 75–80% after 28 weeks of pregnancy [1]. In our study, in-utero transmission was only documented in one infant who had a positive HIV PCR three days after birth (Table 2), whose mother only presented for ANC in the third trimester. Studies that assessed MTCT among pregnant women who received HAART during ANC period, from 26 or 28 weeks’ gestation through weaning at 6 months post-partum, found that majority of HIV infections were transmitted in-utero [41, 42]. A recent SA study reported a rate of 245 in-utero paediatric HIV infections per 100 000 live births nationally, and this ranged between 263–294 infections per 100 000 live births in the Tshwane district [11]. This shows that it is important for pregnant women to present early for ANC, within the first trimester, in order to maximize the benefits of option B+ strategy.

### Impact of improving HIV testing in ANC clinics

Without intervention, the overall risk of MTCT of HIV is up to 40% among infants who are breastfed [1]. This risk is assessed by infant HIV testing at different time-points after birth. HIV PCR test at birth assesses in-utero transmission, while transmission through breastmilk is assessed during or at cessation of breastfeeding [41, 42]. In our study there was a 9.4% transmission rate, which is difficult to compare to other studies as there was no single time-point where all infants had HIV PCR results. HIV testing in this study was affected by changes in SA PMTCT guidelines as the first infant PCR test was performed at 6 weeks after birth at the time option B+ was introduced in March 2013 [7]. Infant HIV testing schedule, however, changed in June 2015 when birth PCR test was introduced [21] (Table 2). It is likely that MTCT could have been higher among the study participants had they not participated in this study. Studies that assessed the effectiveness of PMTCT programme, from HIV testing during ANC period to HIV testing and prophylaxis among HIV-exposed infants, found that there was a higher risk of HIV transmission to the infants if some steps of PMTCT programme were missed. HIV misdiagnosis during ANC has been identified as one of the factors that contribute significantly to MTCT [28, 43]. The testing strategy employed in this study would be difficult to incorporate into the HIV testing programme as NAAT is expensive and mostly confined to centralized laboratories. This would be resolved by availability of POC HIV molecular tests; however, these tests are still under evaluation [44, 45].

### HIV testing schedule during ANC period

Following the first ANC visit within the first trimester, guidelines for maternity care in SA recommend follow-up ANC visits at gestations 20, 26–28, 32–34 and 38 weeks, and 41 weeks if still pregnant [46]. Using the recommended 3 monthly HIV retesting during the study period shows that most women in this study should have had at least two HIV tests before delivery if they adhered to the ANC visit schedule [46]. A single false-negative test in a woman who presented for the first ANC visit at 36 weeks of gestation resulted in her not initiated on PMTCT prior to labour (Table 2, #25). She was not tested for HIV during labour at 38 weeks, hence she was only started on PMTCT three weeks post-delivery when she came back for positive NAAT results. This highlights that it could be difficult to offer HIV testing during the labour period.

The current SA HIV testing guidelines, implemented in 2016, recommend retesting during pregnancy at every ANC visit for women who had a negative test at the first ANC visit [18]. These guidelines represent an improvement in HIV testing, but their effectiveness would probably be determined by early presentation for ANC. Shortening the HIV retesting interval in individuals with negative HIV test is supported by our previous publication, which found that follow-up rapid tests were positive at a median interval of 4 weeks in individuals who were initially misdiagnosed [14].

The limitations of this study include a small sample size, and high frequency of loss to follow-up that led to missing data for some participants. HIV infection could not be excluded in some infants who only had one HIV PCR result and were still breastfeeding. There is no unique laboratory identity number in SA public sector laboratories that is used to link patient’s results at different time points; this could have made it difficult to locate some of the infants’ laboratory results. This study is not representative of all HIV-infected women as it assessed vertical transmission only among women who were initially misdiagnosed by rapid HIV tests at the ANC clinics.

## Conclusions

This study showed that improved HIV testing for pregnant women led to earlier linkage to the PMTCT programme. Inaccurate diagnosis of HIV infection at ANC clinics is likely to undermine the efforts of eMTCT as the important opportunity for initiating PMTCT is missed for some women. Late presentation for the first ANC visit poses a serious challenge to the efficacy of PMTCT. This study shows a need to strengthen HIV diagnosis at the ANC clinics and to encourage pregnant women to present earlier for ANC.

## Supporting information

S1 Table Characteristics of participants diagnosed with early or chronic HIV infection. *Initial tests were performed from samples obtained at enrolment (i.e. after a negative rapid HIV test result). HIV viral load (VL) tests were performed first to screen for HIV infection, and all the serology tests were performed later. Follow-up (F/U) VL was only performed for participants who had an initial VL <5000 copies/ml [16]. Pt ID = participant’s study identity, F = female, gen = generation, ELISA = enzyme-linked immunosorbent assay, W. Blot = Western Blot, LAg = limiting antigen, Insuf = insufficient, LT = long term (chronic) infection, --- = not available (participant did not return for follow-up), + = positive,— = negative. Units: HIV VL = copies/ml; p24 antigen = cut-off index (COI); Genscreen ELISA = sample cut-off (S/CO); LAg avidity = normalized optical density (OD-n); LAg avidity <1.5 OD-n = early infection; LAg avidity >1.5 OD-n = LT (chronic) infection. ^¥^ = participant 6738 was previously misclassified as having chronic infection [16], but testing on her follow-up sample revealed low avidity antibodies consistent with early infection; this was confirmed on repeat testing of 6738 sample. P24 antigen, W. Blot and F/U LAg were not performed for the last participants identified with newly diagnosed HIV infection owing to cost limitations. This also applies to the F/U VL for participant 1692, as this was supposed to have been performed according to the diagnostic study protocol [14].
(DOCX)
