# Supplementary material for: Vertical transmission of HIV among pregnant women who initially had false–negative rapid HIV tests in four South African antenatal clinics
Source: PLoS One. 2019 Dec 20;14(12):e0226391. doi: 10.1371/journal.pone.0226391 (PMC6924658; doi:10.1371/journal.pone.0226391)
Supplement: S1 Table — *Initial tests were performed from samples obtained at enrolment (i.e. after a negative rapid HIV test result). HIV viral load (VL) tests were performed first to screen for HIV infection, and all the serology tests were performed later. Follow-up (F/U) VL was only performed for participants who had an initial VL <5000 copies/ml [16]. Pt ID = participant’s study identity, F = female, gen = generation, ELISA = enzyme-linked immunosorbent assay, W. Blot = Western Blot, LAg = limiting antigen, Insuf = insufficient, LT = long term (chronic) infection, --- = not available (participant did not return for follow-up), + = positive,— = negative. Units: HIV VL = copies/ml; p24 antigen = cut-off index (COI); Genscreen ELISA = sample cut-off (S/CO); LAg avidity = normalized optical density (OD-n); LAg avidity <1.5 OD-n = early infection; LAg avidity >1.5 OD-n = LT (chronic) infection. ¥ = participant 6738 was previously misclassified as having chronic infection [16], but testing on her follow-up sample revealed low avidity antibodies consistent with early infection; this was confirmed on repeat testing of 6738 sample. P24 antigen, W. Blot and F/U LAg were not performed for the last participants identified with newly diagnosed HIV infection owing to cost limitations. This also applies to the F/U VL for participant 1692, as this was supposed to have been performed according to the diagnostic study protocol [14]. (DOCX) [file pone.0226391.s001.docx]

S1 Table. Characteristics of participants diagnosed with early or chronic HIV infection

|  |  | **INITIAL TESTS*** | | | | | | **FOLLOW-UP TESTS** | | | | | |
| --- | --- | --- | --- | --- | --- | --- | --- | --- | --- | --- | --- | --- | --- |
| **Pt ID** | **Sex** | **Rapid HIV test** | **HIV VL** | **p24 antigen** | **Genscreen 3^rd^ gen ELISA** | **W. Blot** | **LAg**  **avidity** | **F/U interval (weeks)** | **F/U Rapid HIV test** | **F/U**  **HIV VL** | **F/U LAg**  **avidity** | **HIV**  **Staging** | **Pregnancy** |
| 6638 | F | - | 195105 | - (0.3) | + (4.45) | + | 0.256 | 6 | + |  | 0.437 | Early | Yes |
| 6512 | F | - | 1763 | - (0.3) | + (4.93) | + | 1.312 | 2 | + | 1938 | 1.111 | Early | Yes |
| 6743 | F | - | 27364 | - (0.5) | + (4.48) | - | 0.108 | 7 | + |  | 0.418 | Early | Yes |
| 6582 | F | - | 6216 | INSUF | + (4.81) | + | 0.458 | 6 | + |  | 1.060 | Early | Yes |
| 6727 | F | - | 4874 | + (2.6) | + (4.48) | + | 0.234 | 2 | + | 1970 | 0.258 | Early | Yes |
| 6737 | F | - | 2227 | - (0.3) | + (5.95) | + | 0.746 | --- | --- | --- | --- | Early | Yes |
| 2504 | F | - | 37243 | - (0.4) | + (5.95) | + | 1.211 | 2 | + |  | 1.391 | Early | Yes |
| 3469 | F | - | 33274 | + (5.6) | + (5.52) | + | 0.832 | 9 | + |  | 0.763 | Early | Yes |
| 2866 | F | - | 3352 | ND | + (4.83) | ND | 0.114 | --- |  |  | --- | Early | Yes |
| 4631 | F | - | 12412 | ND | + (0.18) | ND | 0.076 | --- |  |  | --- | Early | Yes |
| 6738^¥^ | F | - | 159539 | - (0.37) | + (5.96) | + | 0.079 | 2 | + |  | 0.080 | Early | Yes |
| 5054 | F | - | 27820 | - (0.29) | + (4.54) | + | 4.246 | 2 | + |  | 3.577 | LT | Yes |
| 5067 | F | - | 12675 | INSUF | + (4.22) | + | 3.317 | 8 | + |  | 2.822 | LT | Yes |
| 9915 | F | - | 14100 | + (1.36) | + (5.95) | + | 4.175 | 2 | + |  | 3.341 | LT | Yes |
| 639 | F | - | 6579 | - (0.25) | + (4.63) | + | 3.853 | 4 | + |  | 3.099 | LT | Yes |
| 8828 | F | - | 41500 | - (0.42) | + (5.95) | + | 2.382 | 14 | + |  | 2.849 | LT | Yes |
| 2678 | F | - | 222853 | + (11.13) | + (4.69) | + | 3.639 | 4 | + |  | 3.135 | LT | Yes |
| 9895 | F | - | 4880 | - (0.29) | + (4.21) | + | 4.305 | 6 | + | 7873 | 3.480 | LT | Yes |
| 9986 | F | - | 97600 | - (0.92) | + (4.75) | + | 3.700 | 2 | + |  | 3.093 | LT | Yes |
| 843 | F | - | 29712 | - (0.29) | + (4.63) | + | 2.252 | 5 | + |  | 2.463 | LT | Yes |
| 6990 | F | - | 17536 | - (0.33) | + (5.96) | + | 3.945 | 6 | + |  | 3.233 | LT | Yes |
| 2340 | F | - | 14490 | - (0.36) | + (4.63) | + | 2.850 | --- | --- |  | --- | LT | Yes |
| 6709 | F | - | 53 | - (0.34) | + (4.48) | + | 3.162 | --- | --- | --- | --- | LT | Yes |
| 6748 | F | - | 932 | - (0.29) | + (4.68) | + | 4.085 | 2 | + | 1707 | 3.324 | LT | Yes |
| 6671 | F | - | 14072 | - (0.23) | + (4.48) | + | 4.083 | 3 | + |  |  | LT | Yes |
| 6380 | F | - | 11073 | - (0.23) | + (4.62) | + | 4.763 | 4 | + |  | 3.509 | LT | Yes |
| 6557 | F | - | 614 | - (0.33) | + (4.41) | + | 4.049 | 4 | + | 265 | 3.593 | LT | Yes |
| 6565 | F | - | 5670 | - (0.37) | + (4.32) | + | 3.091 | 3 | + |  | 2.969 | LT | Yes |
| 6596 | F | - | 3873 | - (0.36) | + (5.95) | + | 4.385 | 3 | + | 1087 | 3.519 | LT | Yes |
| 6640 | F | - | 3074 | - (0.34) | + (4.48) | + | 2.824 | 5 | + | 9887 | 2.864 | LT | Yes |
| 6649 | F | - | 21051 | - (0.28) | + (5.95) | + | 3.804 | 2 | + |  | 2.867 | LT | Yes |
| 1067 | F | - | 1779 | - (0.37) | + (4.75) | + | 4.088 | 2 | + | 2574 | 3.470 | LT | Yes |
| 921 | F | - | 9781 | - (0.28) | + (5.04) | + | 4.553 | 7 | + |  | 3.607 | LT | Yes |
| 3869 | F | - | 217372 | - (0.31) | + (5.52) | + | 3.716 | 4 | + |  | 3.264 | LT | Yes |
| 3912 | F | - | 32008 | - (0.29) | + (5.52) | + | 2.314 | 8 | + |  | 2.079 | LT | Yes |
| 3920 | F | - | 66694 | - (0.44) | + (4.68) | + | 4.375 | 8 | + |  | 3.410 | LT | Yes |
| 3880 | F | - | 7505 | - (0.30) | + (4.62) | + | 3.840 | 3 | + |  | 3.206 | LT | Yes |
| 3935 | F | - | 242663 | + (21.77) | + (5.95) | + | 1.833 | 8 | + |  | 2.157 | LT | Yes |
| 1117 | F | - | 153 | - (0.31) | + (5.95) | + | 3.315 | 2 | + | 629 | 3.249 | LT | Yes |
| 1121 | F | - | 80287 | - (0.34) | + (5.95) | + | 3.634 | 2 | + |  | 3.483 | LT | Yes |
| 3474 | F | - | 16510 | - (0.32) | + (4.91) | + | 2.078 | 12 | + |  | 2.828 | LT | Yes |
| 1475 | F | - | 44450 | + (1.17) | + (4.92) | + | 4.241 | 9 | + |  | 3.628 | LT | Yes |
| 3387 | F | - | 79597 | ND | + (4.57) | ND | 3.325 | --- | --- |  | --- | LT | Yes |
| 3253 | F | - | 89787 | ND | + (5.21) | ND | 2.244 | 3 | + |  | ND | LT | Yes |
| 1692 | F | - | 396 | ND | + (5.23) | ND | 2.409 | 3 | + | ND | ND | LT | Yes |
| 3606 | F | - | 15940 | ND | + (5.23) | ND | 3.446 | --- | --- |  | --- | LT | Yes |
| 1213 | F | - | 32604 | ND | + (4.92) | ND | 2.473 | --- | --- |  | --- | LT | Yes |
| 3910 | F | - | 28938 | ND | + (5.05) | ND | 2.958 | 3 | + |  | ND | LT | Yes |

*Initial tests were performed from samples obtained at enrolment (i.e. after a negative rapid HIV test result). HIV viral load (VL) tests were performed first to screen for HIV infection, and all the serology tests were performed later. Follow-up (F/U) VL was only performed for participants who had an initial VL <5000 copies/ml [16]. Pt ID = participant’s study identity, F = female, gen = generation, ELISA = enzyme-linked immunosorbent assay, W. Blot = Western Blot, LAg = limiting antigen, Insuf = insufficient, LT = long term (chronic) infection, --- = not available (participant did not return for follow-up), + = positive, - = negative. Units: HIV VL = copies/ml; p24 antigen = cut-off index (COI); Genscreen ELISA = sample cut-off (S/CO); LAg avidity = normalized optical density (OD-n); LAg avidity <1.5 OD-n = early infection; LAg avidity >1.5 OD-n = LT (chronic) infection. ^¥^ = participant 6738 was previously misclassified as having chronic infection [16], but testing on her follow-up sample revealed low avidity antibodies consistent with early infection; this was confirmed on repeat testing of 6738 sample. P24 antigen, W. Blot and F/U LAg were not performed for the last participants identified with newly diagnosed HIV infection owing to cost limitations. This also applies to the F/U VL for participant 1692, as this was supposed to have been performed according to the diagnostic study protocol [14].
